# Loss of the V-ATPase B1 Subunit Isoform Expressed in Non-Neuronal Cells of the Mouse Olfactory Epithelium Impairs Olfactory Function

**DOI:** 10.1371/journal.pone.0045395

**Published:** 2012-09-20

**Authors:** Teodor G. Păunescu, Steven Rodriguez, Eric Benz, Mary McKee, Robert Tyszkowski, Mark W. Albers, Dennis Brown

**Affiliations:** 1 MGH Center for Systems Biology, Program in Membrane Biology and Division of Nephrology, Massachusetts General Hospital, Boston, Massachusetts, United States of America; 2 Harvard Medical School, Boston, Massachusetts, United States of America; 3 MassGeneral Institute for Neurodegenerative Disease, Charlestown, Massachusetts, United States of America; Rutgers University, United States of America

## Abstract

The vacuolar proton-pumping ATPase (V-ATPase) is the main mediator of intracellular organelle acidification and also regulates transmembrane proton (H^+^) secretion, which is necessary for an array of physiological functions fulfilled by organs such as the kidney, male reproductive tract, lung, bone, and ear. In this study we characterize expression of the V-ATPase in the main olfactory epithelium of the mouse, as well as a functional role for the V-ATPase in odor detection. We report that the V-ATPase localizes to the apical membrane microvilli of olfactory sustentacular cells and to the basolateral membrane of microvillar cells. Plasma membrane V-ATPases containing the B1 subunit isoform are not detected in olfactory sensory neurons or in the olfactory bulb. This precise localization of expression affords the opportunity to ascertain the functional relevance of V-ATPase expression upon innate, odor-evoked behaviors in B1-deficient mice. This animal model exhibits diminished innate avoidance behavior (revealed as a decrease in freezing time and an increase in the number of sniffs in the presence of trimethyl-thiazoline) and diminished innate appetitive behavior (a decrease in time spent investigating the urine of the opposite sex). We conclude that V-ATPase-mediated H^+^ secretion in the olfactory epithelium is required for optimal olfactory function.

## Introduction

The vacuolar proton-translocating ATPase (vacuolar, or V-type, H^+^ATPase, or V-ATPase) plays an essential role in numerous vesicle trafficking processes. When expressed at the plasma membrane, the V-ATPase performs various cell type-specific functions: in renal collecting duct A-type intercalated cells, it is the main mediator of acid secretion by the kidney, thus maintaining body acid-base homeostasis [1], [2], [3], [4]; in epididymal clear cells, it is responsible for the acidification of the luminal fluid that is necessary for sperm storage and maturation [5], [6], [7]; in neutrophils and macrophages, it regulates cytosolic pH [8], [9]; in interdental cells of the inner ear, it is involved in regulating the pH of the endolymph [10], [11], [12]; and in osteoclasts, it is required for bone resorption [13], [14], [15], [16].

Given that the olfactory epithelium (OE) has been implicated in acid-base sensing and/or regulation [17] and that this tissue expresses carbonic anhydrase [17], [18], [19], [20], [21], an enzyme which is also highly expressed in H^+^-secreting cells in other tissues, we investigated whether the V-ATPase is expressed in this tissue. We detected various subunits of this enzyme in cells of the main OE (MOE) and of the vomeronasal organ (VNO) in the mouse [22]. Importantly, one of the V-ATPase subunits that we detected in the OE was the 56-kDa B1 subunit isoform, a marker of specialized H^+^-secreting cells in other organs, such as the kidney, lung, male reproductive tract, eye and ear [5], [12], [23], [24], [25], [26], [27]. We reported that the B1 subunit of the V-ATPase localizes to the apical microvilli of sustentacular cells (SCs) and to the lateral membrane in a subset of other epithelial cells of the mouse OE [22].

Based on the involvement of SCs in ion [28], [29], [30], [31], salt, and possibly water [28], [32] transport in the neuroepithelial mucous layer (NML), we hypothesized that V-ATPase-mediated H^+^ secretion may confer on SCs the role of acid-base regulators of the extracellular milieu in the OE. Consequently, the physiological significance of V-ATPase expression in the OE was assumed to involve acidifying the NML, which in turn could be important for sensitivity to odorants [22] - reminiscent of the way in which the mucosal concentration of various ions, including Na^+^, K^+^, and Ca^2+^, affects the sensitivity of odor detection [33]. It has been previously postulated not only that the V-ATPase is involved in regulating the pH of the NML but also that it may power other ion transport mechanisms and thus regulate the ionic balance of this layer [29]. Since in many mammals the NML contains odorant binding proteins [34], both odor discrimination and odor intensity could be dependent on pH, and hence on V-ATPase activity.

Absorption of various substances in the nasal mucosa depends on the pH of the formulation, exhibiting an increase in bioavailability at lower pH values – e.g. for azetirelin [35] and secretin [36] in rats and insulin in dogs [37]. For dairy products in which volatile fatty acids are important flavor compounds, the pH of the aqueous phase influences perception by affecting the dissociation equilibria of these acids [38]. Nasal toxicity varies depending on the thickness and pH of the NML, among other factors [39]. Vanilloid receptors in sensory neurons respond to capsaicin in a pH-dependent manner, i.e. the potency of capsaicin increases at lower pH values [40]. Moreover, V-ATPase alongside other putative membrane transporters is critical for maintaining intracellular pH [41], which may impact a wide array of physiologic and metabolic processes.

In the present study, we characterize the expression pattern of the V-ATPase in the MOE with greater resolution using immunohistochemical methods followed by fluorescence, confocal and electron microscopy. In addition, we investigate the involvement of this enzyme in olfactory function through behavioral studies in mice that are null for the critical B1 subunit of the V-ATPase.

## Methods

### Antibodies

To characterize V-ATPase expression in the mouse olfactory epithelium, we used previously described affinity-purified polyclonal antibodies raised in rabbit against mouse ATP6V1A (the V-ATPase 70-kDa “A” subunit) [42] and ATP6V1B1 (the V-ATPase 56-kDa “B1” subunit isoform) [22] and in chicken against mouse ATP6V1B1 [43].

We used the following commercially available antibodies as olfactory markers: a goat polyclonal antibody against olfactory marker protein (OMP) (WAKO Chemicals USA, Richmond, VA), a rabbit polyclonal antibody against cytokeratin-18 (CK-18) (Abcam, Cambridge, MA), a rabbit polyclonal antibody against cyclic nucleotide gated cation channel 2 (CNGA2) (Alomone Labs, Jerusalem, Israel), a mouse monoclonal anti-tubulin antibody and a rat monoclonal anti-L-CAM/E-cadherin antibody (Sigma-Aldrich, St. Louis, MO).

The secondary antibodies used for this study were indocarbocyanine (Cy3)- or fluorescein isothiocyanate (FITC)-conjugated donkey anti-rabbit, Cy3-conjugated donkey anti-chicken and anti-rat, and Cy3- and FITC-conjugated donkey anti-goat antibodies purchased from Jackson ImmunoResearch Laboratories (West Grove, PA).

### Tissue Preparation and Immunohistochemistry

Olfactory epithelial tissues were prepared and subjected to immunohistochemistry protocols as previously reported [22]. Mouse pups (2-weeks) or adult mice, wild type (Atp6v1b1+/+) (C57BL6, Jackson Laboratory, Bar Harbor, ME) and B1 V-ATPase-deficient (Atp6v1b1−/−, B1-deficient) [44] were deeply anesthetized with 200 mg/kg body weight i.p. pentobarbital sodium (Nembutal, Abbott Laboratories, Abbott Park, IL). The head was dissected and fixed with 4% paraformaldehyde (Electron Microscopy Sciences, Hatfield, PA) in phosphate-buffered saline (PBS) for 4 h at room temperature and subsequently overnight at 4°C. All animal studies were approved by the Massachusetts General Hospital Subcommittee on Research Animal Care, in accordance with the NIH, Department of Agriculture, and AAALAC requirements.

Fixed tissues were extensively washed in PBS, and pup tissues were subsequently stored at 4°C in PBS containing 0.02% sodium azide until use. Adult tissues were stored in a similar way after being decalcified using Immunocal (Decal Chemical Corp., Tallman, NY) or Cal-Ex (Fisher Scientific, Pittsburgh, PA) per the manufacturer’s recommendations. As discussed previously, cryosectioning of pup tissues did not require prior decalcification [22]. For cryosectioning, tissues were cryoprotected in PBS containing 0.9 M sucrose, embedded in Tissue-Tek OCT compound 4583 (Sakura Finetek USA, Inc., Torrance, CA), and frozen at −20°C as previously described [45], [46]. Sections were cut on a Leica CM3050 S cryostat (Leica Microsystems Inc., Buffalo Grove, IL) at 5 µm for regular immunohistochemistry and at 16 µm for 3-D image reconstruction. Tissue sections were collected onto Superfrost Plus microscope slides (Fisher Scientific), air-dried and stored at 4°C until use. For immunohistochemistry, sections were rehydrated in PBS, treated with sodium dodecyl sulfate (1% wt/vol. in PBS for 4 min) for antigen retrieval [47], washed in PBS, and incubated with 1% bovine serum albumin in PBS for 10 min followed by the primary antibody diluted in Dako antibody diluent (Dako, Carpinteria, CA) for 90 min at room temperature as previously described [22], [25], [46]. Slides were then washed and incubated for 1 h with the respective secondary antibody, rinsed in PBS, and mounted in Vectashield medium (Vector Laboratories, Burlingame, CA) containing 4,6-diamidino-2-phenylindole (DAPI). For dual immunofluorescence staining, the two primary antibodies were applied either simultaneously (followed by the secondary antibody mixture) or sequentially, with each primary antibody followed by the respective secondary antibody. We found these two approaches to yield similar results.

Digital images were acquired using a Nikon Eclipse 80i epifluorescence microscope (Nikon Instruments, Melville, NY) fitted with an Orca 100 CCD camera (Hamamatsu, Bridgewater, NJ) and analyzed using IPLab version 3.2.4 image processing software (Scanalytics, Inc., Fairfax, VA) [22], [25].

For confocal laser scanning microscopy, imaging was performed on a Zeiss Radiance 2000 confocal microscopy system (Carl Zeiss Microimaging, Thornwood, NY) using LaserSharp 2000 version 4.1 software. 3-D image reconstruction was performed using Volocity software (Improvision, Waltham, MA) on stacks of 60 images collected at 0.25 µm intervals along the z-axis as previously described [43]. Images were subsequently imported as 3-D projections into Adobe Photoshop version 6.0 image-editing software (Adobe Systems Inc., San Jose, CA). Final figures for epifluorescence and confocal microscopy were produced using the same software.

### Immunogold Electron Microscopy

Tissues were processed and 50 µm thick sections were cut as described above. Tissue sections were floated off in PBS, dehydrated, embedded, and polymerized at 50°C in LR White resin (Electron Microscopy Sciences) as previously described [43], [48]. Thin (70–80 nm) sections were cut on a Leica EM UC7 ultramicrotome (Leica Microsystems) and incubated on drops of rabbit primary antibody against V-ATPase A-subunit diluted in Dako diluent for 2 h at room temperature, rinsed in PBS, and then incubated on drops of goat anti-rabbit IgG coupled to 15 nm gold particles (Ted Pella, Redding, CA) for 1 h at room temperature. After several rinses with distilled water, the grids were post-stained on drops of 2% uranyl acetate for 5 min, rinsed again in distilled water, and dried. For dual immunostaining, grids were incubated with a mixture of primary antibodies against V-ATPase A-subunit and tubulin. The secondary antibodies were also applied simultaneously, goat anti-rabbit IgG coupled to 15 nm gold particles and respectively anti-mouse IgG coupled to 10 nm gold particles (Ted Pella). Sections were examined in a JEM-1011 transmission electron microscope (JEOL Ltd., Tokyo, Japan) at 80 kV, and images were acquired using an AMT XR60 digital imaging system (Advanced Microscopy Techniques, Danvers, MA). Final figures were produced using Adobe Photoshop as described above.

### Home-cage Tests to Assess the Ability of Female Mice to Detect Male Urinary Odors

We collected urine from adult heterozygous (Atp6v1b1+/−) male mice and performed home-cage tests on sexually naive adult wild-type and B1-deficient females in vaginal proestrus or estrus, similar to previously described habituation-dishabituation tests [49], [50], [51]. Each wild-type or B1-deficient female received two 2-min. presentations of distilled water followed by two 2-min. presentations of male urine. These stimuli were presented at 4-min. intervals, by pipetting 10 µl of fluid on a 2 cm^2^ piece of filter paper taped to a plastic weigh boat [49], [50]. Stimulus investigation (sniffing) is defined as an inhalation directed at the filter paper with the tip of the nose within 1 cm of the filter paper. Animal behavior was followed in real time by visual observation, and stimulus investigation time was recorded using a programmable timer (Fisher Scientific). Unpaired t-tests were used to analyze the data.

### Avoidance of a Predator Odor

We investigated the innate avoidance behavior that mice are known to display toward predator odors [52], [53] by using a test cage divided by a curtain into two compartments [54]. Specifically, trimethyl-thiazoline (TMT), a volatile odor isolated from fox feces, was used. Briefly, the experiment was performed in the dark during the nocturnal phase of the light cycle. The behavioral arena was placed in a chemical hood. Mice were habituated to the arena for 3×10 min with blank filter papers. After 7 min. of the third habituation, the filter papers were swapped with identical filter papers with 20 µL of water or 4% TMT dissolved in water, respectively. The remaining three minutes were video recorded and scored in a blinded fashion. Scoring included summing the accumulated time that the mouse spent freezing, defined as absence of movement save for thoracic oscillations consistent with breathing, and the number of sniffs within a 1 cm radius of each filter paper during the three minute epoch. Since the TMT concentration is suprathreshold, most avoidance and freezing behavior was initiated at distances much greater than 1 cm from the filter paper. While the mice had direct nasal access to the filter paper, we did not observe that any mouse directly contacted filter paper; thus, we interpret this phenotype as mediated by the main olfactory system and not the VNO. One-way analysis of variance and unpaired t-tests were used to analyze the data.

### Mucosal pH Measurements

Whole olfactory epithelia from adult wild-type and B1-deficient female mice were dissected and cut with a razor blade into right, septal, and left parts. pH readings were taken from the luminal surface of each olfactory epithelial part using micro pH strips (pHydrion MicroFine, Micro Essential Laboratory, Brooklyn, NY) in the pH range of 6.0 to 8.0. Each micro pH strip was read by two different investigators unaware of the mouse genotype and averaged; the values from the three different regions were further averaged to yield the recorded mucosal pH value for the respective animal. For any given measurement point, a majority of the two investigators’ readings were identical, and the largest difference between the two was 0.2 pH units. For any given animal, the differences between olfactory epithelial parts, if any, were within 0.2 pH units, with the exception of one mouse in which the differences ranged between 0.1 and 0.3 pH units. Unpaired t-tests were used for the statistical analysis of the data.

## Results and Discussion

### Localization of the V-ATPase in the Mouse Main Olfactory Epithelium (MOE)

**Figure 1:**
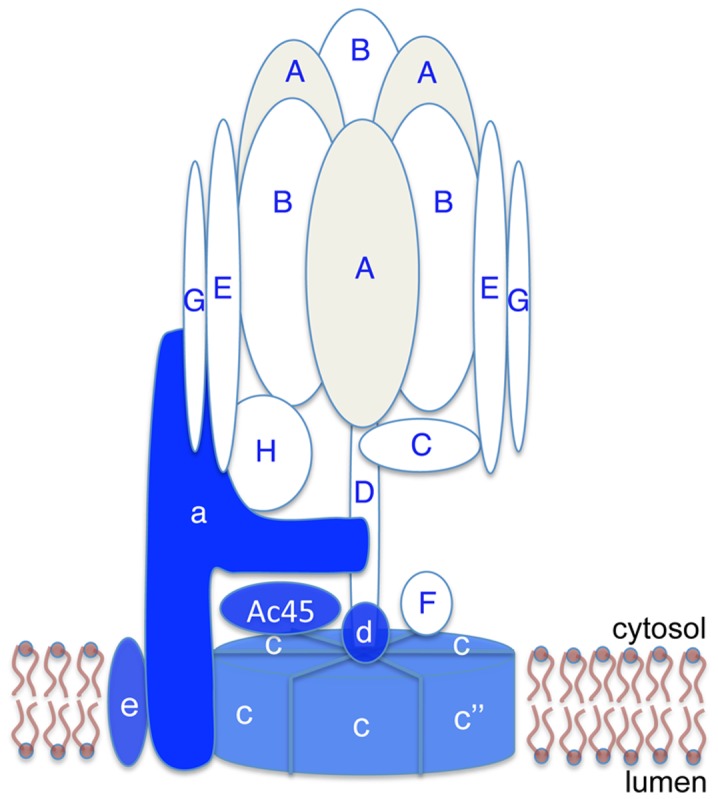
V-ATPase localization in the mouse olfactory epithelium. (A) A schematic diagram showing the subunit composition of the V-ATPase. The cytosolic V_1_ domain is composed of subunits A through H (shown in white or light gray, marked with blue letters). The transmembrane V_0_ domain is composed of subunits a, c, c” (or b), d, e, and Ac45 (shown in blue, marked with white letters). Some of the subunit interactions are putative. (B) Section from a 3-D image reconstruction showing that B1 V-ATPase (red) localizes to the microvilli of olfactory sustentacular cells in a 2-week old female mouse pup. Apical cilia of olfactory sensory neurons are immunostained for CNGA2 (green). DAPI (blue) stains cell nuclei. Bar = 30 µm.

The V-ATPase is a complex enzyme, composed of at least 14 subunits assembled into a transmembrane, proton- (H^+^-) translocating V_0_ sector and a cytosolic, catalytic V_1_ domain (Fig. 1) [55], [56], [57], [58]. In mammalian tissues the 56-kDa B subunit of the enzyme is expressed as two isoforms, B1 and B2. B1 is the B subunit isoform generally associated with regulated transmembrane H^+^ secretion in epithelia [5], [23], [24], [25], and localizes at high levels to the cell plasma membrane and to the submembrane domain. B2 is to a lesser extent associated with the cell plasma membrane, but predominantly with the membranes of intracellular organelles [22], [25].

**Figure 2:**
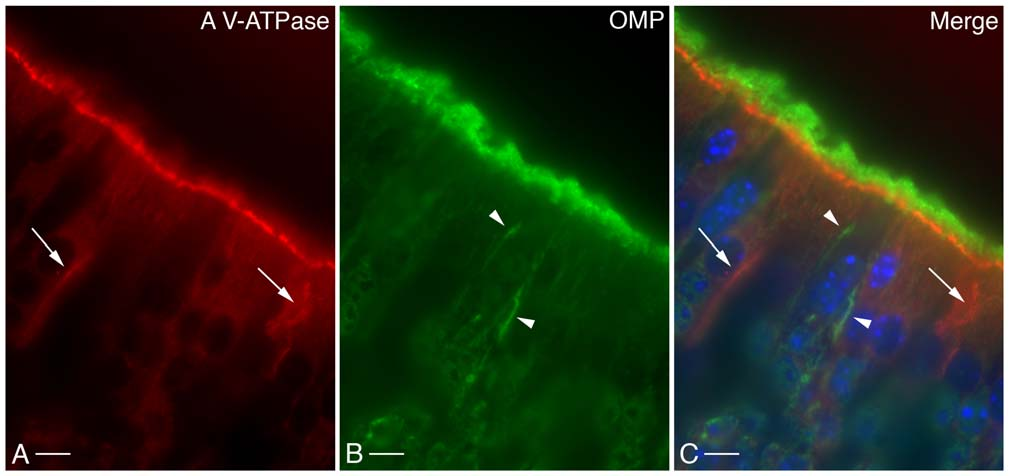
Immunocytochemical localization of the V-ATPase A subunit (A, red) in the OE of an adult male mouse decalcified with ImmunoCal. The V-ATPase is expressed on the apical microvilli of SCs and on the basolateral membrane of a subpopulation of cells (arrows). Olfactory marker protein (OMP, B, green) localizes to the OSN cilia and to the basolateral membrane of OE cells (arrowheads) which, as revealed by the merge panel (C), do not coincide with the cells that express B1 in this membrane domain. DAPI (blue) stains cell nuclei. Bar = 10 µm.

V-ATPase expression and localization in the MOE of adult and juvenile mice were investigated by immunohistochemistry. The results were similar in the two age groups [22]. The B1 subunit isoform localizes mostly to the apical microvilli of SCs, as we originally described [22]. To confirm and more thoroughly examine this localization pattern, we performed dual immunofluorescence staining for V-ATPase and the olfactory sensory neuron (OSN) markers, the cyclic nucleotide gated cation channel 2 (CNGA2) and the olfactory marker protein (OMP). Our results show that the V-ATPase B1 subunit does not colocalize with CNGA2, but instead is detected in a layer that does not extend into the lumen as far as the region occupied by this OSN marker. Similarly, the A subunit of the V-ATPase (Fig. 2A), which is the subunit that most closely associates with the B subunit within the complex V-ATPase structure (Fig. 1), does not colocalize with OMP (Fig. 2B). OMP-associated immunostaining is located more apically relative to the V-ATPase signal (Fig. 2C). These results are consistent with CNGA2 and OMP staining OSN cilia, whereas anti-V-ATPase antibodies stain SC microvilli.

**Figure 3:**
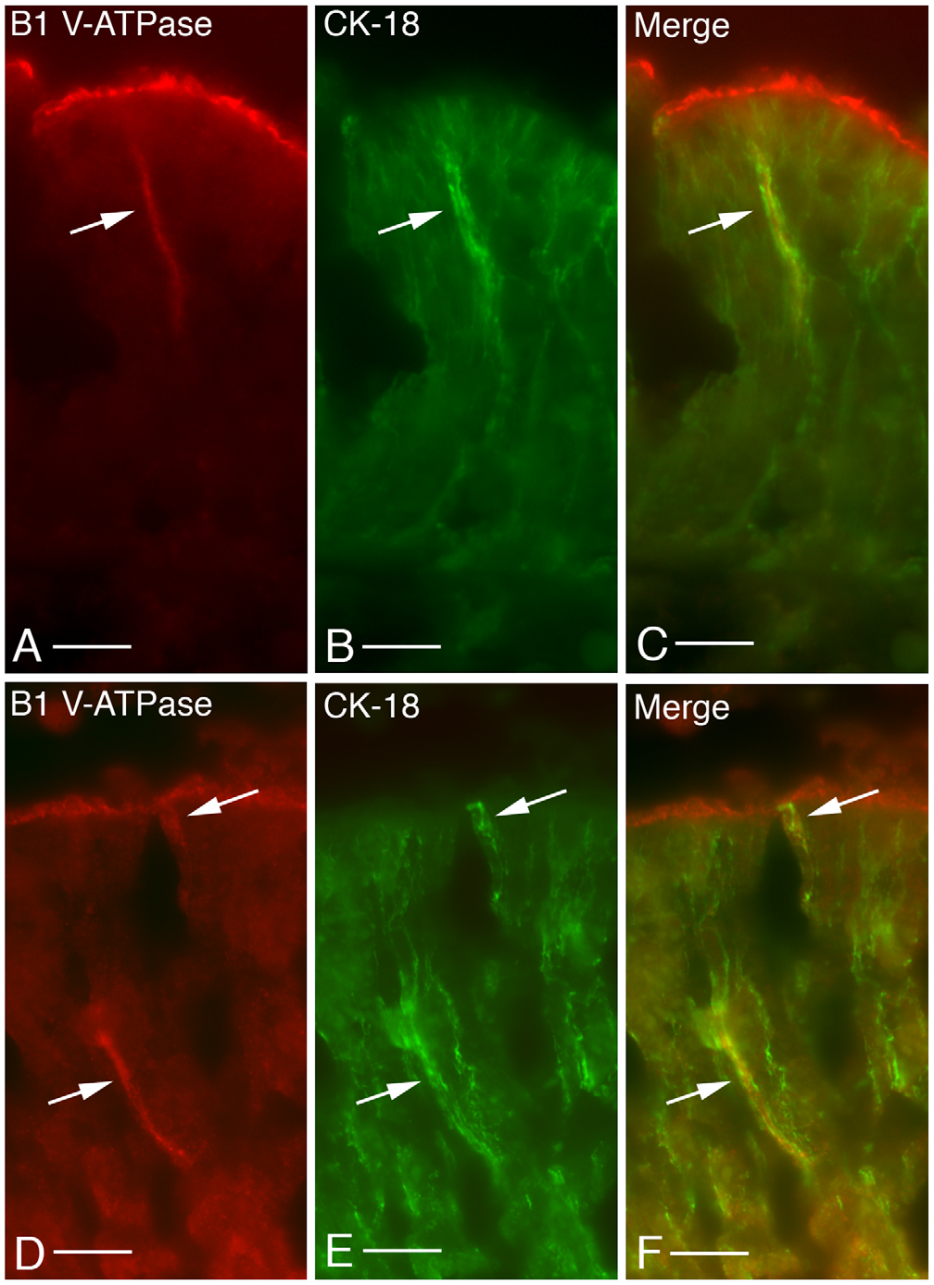
Dual immunofluorescence labeling for the V-ATPase B1 subunit isoform (red) and cytokeratin-18 (CK-18, green) in the olfactory mucosa of an adult male mouse decalcified with Cal-Ex. As also shown in the previous figure for the A subunit, B1 localizes to the apical membrane microvilli of SCs and to the basolateral membrane of a subset of OE cells (A and D, arrows). Interestingly, these cells also exhibit basolateral membrane staining for CK-18 (B and E, arrows). The merge panels (C and F) confirm the co-expression of the V-ATPase B1 subunit and CK-18. Bar = 20 µm.

Besides its SC localization, the V-ATPase was also detected in a subset of cells of the mouse MOE, where it localizes to the basolateral plasma membrane domain [22]. Given the relatively low density of these cells, their location within the MOE and their morphology, we determined that they are most likely not SCs. Dual immunostaining with the V-ATPase A (Fig. 2) and B1 subunit (data not shown) and OMP (Fig. 2) or CNGA2 (data not shown) also rules out the possibility that these cells may be OSNs, as previously presumed [22]. However, dual immunostaining with antibodies against the V-ATPase B1 subunit isoform and cytokeratin-18 (CK-18, Fig. 3) or L-CAM/E-cadherin (data not shown) reveals that the cells with basolateral membrane staining for V-ATPase also express these markers. Taken together, these data allow us to identify this epithelial cell subpopulation as microvillar cells, as previously described in the literature [59], [60].

**Figure 4:**
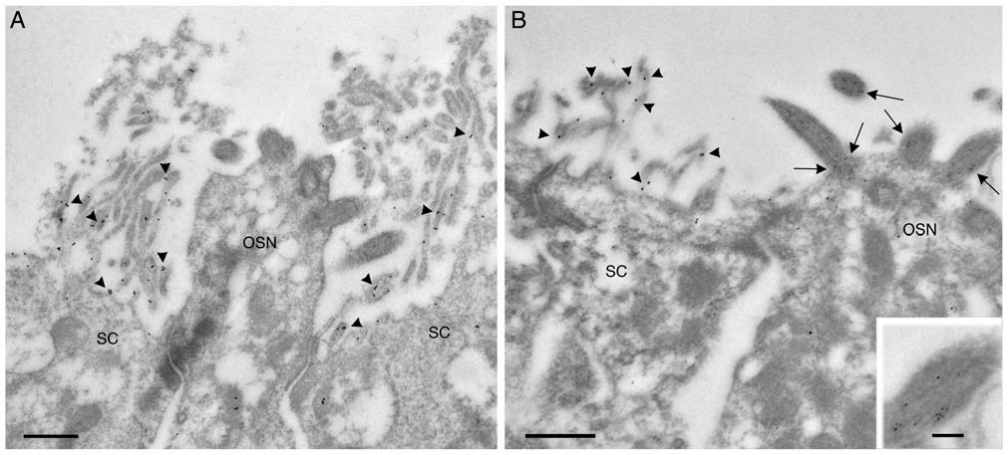
Electron micrographs of the olfactory epithelium from a 2-week old female mouse pup. The V-ATPase A subunit (panel A, arrowheads) is expressed at high levels in the microvilli of sustentacular cells (SC), but is minimally present in the olfactory sensory neurons (OSN), where it localizes to the cytosolic domain. Panel B shows the same expression pattern for the V-ATPase (arrowheads, 15 nm gold particles), whereas tubulin (arrows, 10 nm gold particles) localization is restricted to OSN cilia (shown at higher magnification in inset). Bar = 0.5 µm. Inset bar = 0.1 µm.

The cellular and subcellular localization of V-ATPase in the MOE was further investigated using immunogold electron microscopy (IEM). We found the A subunit of the V-ATPase to be expressed at high levels on the microvilli of olfactory SCs, confirming the immunofluorescence results presented above (Figs. 2–3). To a lesser extent, the A subunit also localized in the cytosolic domain of SCs and OSNs, consistent with the well known presence of A- (but not B1-) containing V-ATPases on the membrane of intracellular organelles in all cells [2], [3], [61]. As expected, tubulin localized exclusively to OSN cilia and not to SCs (Fig. 4, see also higher magnification inset).

Consequently, the V-ATPase, including its B1 subunit isoform, which is a hallmark of regulated transmembrane H^+^ secretion, localizes to the apical membrane microvilli of olfactory sustentacular cells, but not to olfactory sensory neurons. The B1 subunit is also not expressed in the olfactory bulb at detectable levels by immunohistochemistry (T.G. Păunescu, unpublished observations) or in the brain in general, as previously determined by immunoblotting [24]. Brain V-ATPases typically contain the alternate B2 isoform and localize to the membranes of intracellular organelles.

### Diminished Olfactory Function in B1-deficient Mice as Revealed by Home Cage Tests

**Figure 5:**
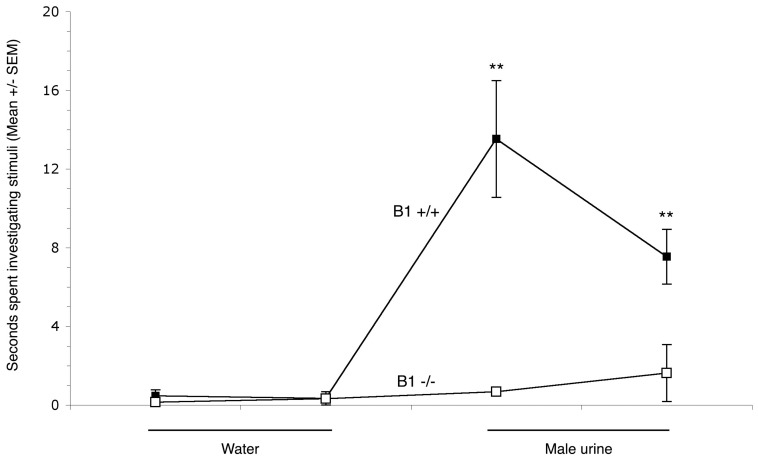
Home-cage tests assessing behavior of wild-type and B1-deficient female mice in investigating urine of heterozygous (Atp6v1b1+/−) male mouse urine. Water presentations revealed no significant difference between wild type and B1-deficient mice or between presentations. Both urine presentations showed that wild-type mice spent significantly more time investigating the urine of the opposite sex than Atp6v1b1−/− mice (n = 6). Data are shown as mean ± SEM (**, p<0.01).

As reviewed above, plasma membrane V-ATPases containing the B1 subunit isoform fulfill cell type-specific roles in all tissues and cells in which the enzyme has been detected. We, therefore, investigated the possible role of this enzyme in the OE. Home-cage tests performed on female wild type and B1-deficient mice with distilled water showed no significant difference between the two groups of animals or between presentations (all p values >0.05, n = 5 wild-type and n = 5 B1-deficient mice), as shown in Fig. 5. However, male urine presentations revealed that wild-type mice spent significantly more time investigating the urine of the opposite sex than B1-deficient mice (p = 0.001 for the first presentation and p = 0.009 for the second stimulus). Interestingly, while urine investigation time declined for the second exposure in wild-type mice (p<0.05), this parameter showed a slightly increasing trend in B1-deficient animals.

The nature of this experiment does not preclude direct contact of urine with the nose and hence involvement of the VNO in generating the perception. However, we still interpret the observed deficit as arising from altered epithelial physiology since V-ATPases containing the B1 subunit isoform are also expressed in the VNO [22].

### Diminished Olfactory Function in B1-deficient Mice as Revealed by Innate Avoidance Behavior Tests

**Figure 6:**
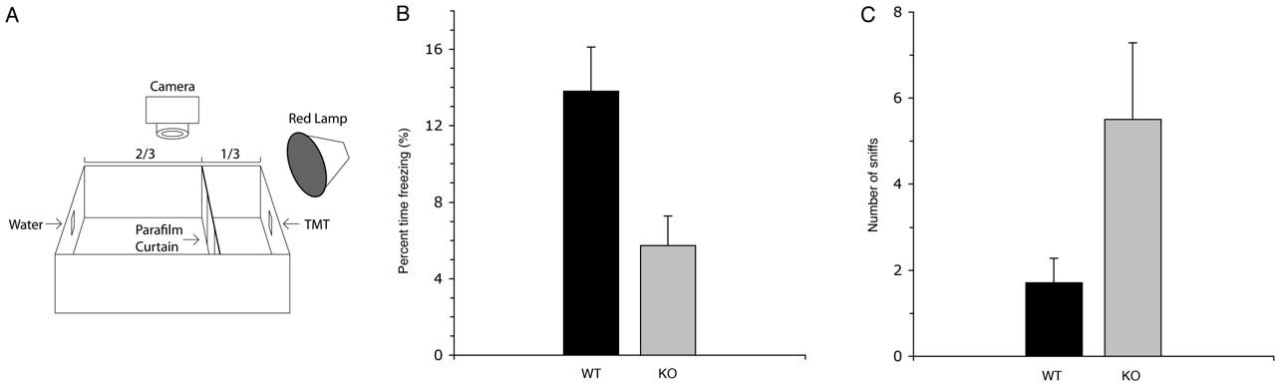
Reduced odor-evoked freezing by a predator odor. (A) A schematic diagram of the behavioral arena is depicted. (B) The percentage of time freezing in adult male B1-deficient mice (“KO”, n = 7) in the presence of the predator odor trimethyl-thiazoline (TMT) is less than half the time recorded in wild-type mice (“WT”, n = 6). Data are shown as mean ± SEM (p = 0.02). (C) Increased investigation of TMT in the V-ATPase B1-deficient mice: The number of individual sniffs of TMT is increased in adult male B1-null mice (“KO”, black bar) relative to wild-type mice (“WT”, gray bar). Data is shown as mean ± SEM (p = 0.05) and confirm the blunted response to TMT in B1-deficient mice compared to their wild type counterparts.

The restricted expression pattern of the V-ATPase to the non-neuronal cells in the OE affords the opportunity to assess the functional role of this protein on olfactory function by surveying behavioral responses to odors isolated from predators with innate behavioral responses. The behavioral arena (Fig. 6A) consisted of a test cage divided by a curtain into two compartments, as described in “Methods”. Adult B1-deficient male mice displayed a blunted response to trimethyl-thiazoline (TMT) compared to their wild type counterparts. The freezing behavior in the two groups of animals was similar in the absence of TMT (p = 0.12, data not shown), whereas the percentage of time spent freezing by B1-deficient mice in the presence of TMT was less than half the time recorded in wild-type animals (p = 0.02, n = 7 wild-type and n = 6 B1-deficient mice) (Fig. 6B). Also consistent with an olfactory deficit, the number of sniffs towards the TMT stimulus was higher in B1-deficient mice compared to wild type controls (p = 0.05) (Fig. 6C, Movie S1 and Movie S2).

In conclusion, our functional studies on mice deficient in the B1 subunit isoform of the V-ATPase [44], [45] revealed diminished innate avoidance behavior, measured as the number of TMT sniffs and time spent exhibiting freezing behavior in the presence of this predator odor, as well as the fact that they spent less time investigating the urine of the opposite sex in comparison to their wild-type counterparts. Female B1-deficient mice are less attracted to male specific urine and male B1-deficient mice avoid less and freeze less in response to a repulsive odorant. Both phenotypes could arise from either olfactory deficits and/or in central circuits that underlie assignment of valence to olfactory input. Since B1 is not expressed in the brain and is strongly expressed in the OE, we favor the olfactory hypothesis and not the central hypothesis.

Data from behavior tests point towards the involvement of V-ATPase in olfactory function. This finding is also consistent with the subcellular localization of the V-ATPase in wild-type vs. B1-deficient mice, as previously reported [22]. In intercalated cells of renal medullary collecting ducts from B1-deficient mice, V-ATPase activity is reduced to 28–40% of normal baseline values under circumstances where B2-containing V-ATPases are inserted maximally in the apical plasma membrane [45]. However, our preceding study showed that, unlike in these renal H^+^-secreting cells [44], [45], subapical V-ATPases containing the alternative B2 subunit isoform do not undergo a localization shift towards the apical microvilli of sustentacular cells in B1-deficient mice [22] and likely do not, therefore, compensate for the lack of the B1 isoform. Consequently, it should be expected that these animals have a defect in V-ATPase-mediated H^+^ secretion in the OE that negatively impacts their ability to detect urinary odors and odors with innate behavior responses. To investigate this possibility, we measured the pH of the luminal side of the olfactory epithelium directly and found a consistent and statistically significant (p = 0.0006) pH increase in the B1-deficient mice compared to wild-type animals. Mucosal pH averaged 6.758±0.036 in wild-type mice (mean ± SD, n = 4) compared to 7.010±0.012 in B1-deficient animals (mean ± SD, n = 4).

These data are consistent with our hypothesis that olfactory sustentacular cells may act through V-ATPases to regulate the pH of the neuroepithelial mucous layer (NML), which could in turn modulate the sensitivity to odorants [22]. This would not be unprecedented, since the NML concentration of various ions (e.g., Na^+^, K^+^, and Ca^2+^) was previously reported to affect the sensitivity of odor detection [33]. Moreover, the V-ATPase was previously postulated to power other ion transport mechanisms in the olfactory mucosa, thus regulating indirectly its ionic balance [29], as is known to happen in the inner ear [62].

We envisage that the pH of the NML could be important in various mechanisms included in classical models of olfaction, such as: 1) the absorption of odorant molecules from the air phase into the NML (mucus solubility and the equilibrium partition coefficient for the odorant at the air-fluid, i.e. mucus interface potentially being pH-dependent), 2) diffusion of odorants across the mucociliary layer (molecular diffusivity of the odorant presumably being pH-dependent), 3) odorant molecule binding to odorant binding protein, 4) odorant molecule-receptor binding on the OSN membrane and cilia (pH was shown to affect protein-ligand binding), and 5) odorant removal (the dissociation constant and the odorant uptake rate potentially being pH-dependent) [63], [64], [65], [66]. Moreover, some peptides and trace amines have been shown to mediate innate behavioral responses via olfaction [67], [68], [69], [70], [71], which could be modulated by pH.

It is of particular interest that the V-ATPase, and especially its B1 subunit isoform, is highly expressed in microvillar cells, where it localizes to the basolateral plasma membrane domain. As far as we know, there is no consensus on the specific physiological role fulfilled by microvillar cells. Intriguingly, we previously reported that cells exhibiting basolateral V-ATPase staining also express carbonic anhydrase type IV (CA IV) [22]. By analogy with CA II-rich olfactory sensory neurons [18], [20], [72] that were reported to be CO_2_ chemoreceptors [20], [73], we raised the question whether these cells may be involved in modulating the pH of the NML in response to CO_2_ [22]. While this idea is yet to be tested directly, its confirmation would provide a novel, unrecognized physiological role for this insufficiently characterized cell type.

The present study is the first to indicate the relevance of the V-ATPase, and presumably of V-ATPase-mediated proton secretion, in olfactory function. Undoubtedly, further functional and behavioral studies will allow a more comprehensive assessment of the physiological and clinical significance of V-ATPase expression in sustentacular and microvillar cells of the olfactory epithelium. At this point, we can only speculate on such possibilities. For example, modulating olfactory H^+^ secretion could offer the ability of up- or down-regulating the threshold of detection for certain odorants. Moreover, since the NML plays a role as a barrier against inhaled pathogens, and microbial and chemical toxins, regulating mucus pH may be relevant for protection against various specific diseases.

## Supporting Information

Movie S1 Representative video recording showing the behavior of a wild-type mouse three minutes after the TMT (left hand side) and water (right hand side) are introduced into the behavioral arena. A directed sniff is indicated by the “sniff” title that appears in the lower right hand corner after the sniff was scored (n = 1 sniff for the wild-type mouse shown).
(M4V)

Movie S2 A similar video recording showing the behavior of a B1-deficient mouse three minutes after the TMT (left hand side) and water (right hand side) are introduced into the behavioral arena (n = 6 sniffs for the B1-deficient mouse shown). Freezing behavior was also quantified in these representative movies, but is not indicated by titles. The wild-type and B1-deficient mice were tested on the same day.
(M4V)
